# Platelet Adhesion on Commercially Pure Titanium Plates in Vitro II. Immunofluorescence Visualization of PDGF-B, TGFβ1, and PPARγ Released from Activated Adherent Platelets

**DOI:** 10.3390/dj7040109

**Published:** 2019-11-19

**Authors:** Tetsuhiro Tsujino, Akira Takahashi, Taisuke Watanabe, Kazushige Isobe, Yutaka Kitamura, Kazuhiro Okuda, Koh Nakata, Tomoyuki Kawase

**Affiliations:** 1 Tokyo Plastic Dental Society, Kita-ku, Tokyo 114-0002, Japan; tetsudds@gmail.com (T.T.); kaz-iso@tc4.so-net.ne.jp (K.I.); 2 Private Practice, Takatsu-ku, Kawasaki 213-0033, Japan; atakahashihdc@ybb.ne.jp; 3 Division of Anatomy and Cell Biology of the Hard Tissue, Institute of Medicine and Dentistry, Niigata University, 2-5274 Gakkocho-dori, Niigata 951-8514, Japan; watatai@mui.biglobe.ne.jp; 4 Department of Oral and Maxillofacial Surgery, Matsumoto Dental University, 1780 Hirooka-gohara, Shiojiri 339-0781, Japan; shinshu-osic@mbn.nifty.com; 5 Division of Periodontology, Institute of Medicine and Dentistry, Niigata University, Niigata 951-8514, Japan; okuda@dent.niigata-u.ac.jp; 6 Bioscience Medical Research Center, Niigata University Medical and Dental Hospital, Niigata 951-8520, Japan; radical@med.niigata-u.ac.jp; 7 Division of Oral Bioengineering, Institute of Medicine and Dentistry, Niigata University, Niigata 951-8514, Japan

**Keywords:** platelet-rich plasma, titanium, adhesion, platelet-derived growth factors, transforming growth factor β, peroxisome proliferator-activated receptor γ

## Abstract

Recent progress in the industrial development of dental implants has improved their surface bio-affinity, while clinical implantologists attempt to improve it through coating with various compounds, including platelet-rich plasma (PRP) in clinical settings. However, it is poorly understood how PRP acts on titanium surfaces. To validate this surface modification method and demonstrate how platelet-derived soluble biomolecules released from the activated adherent platelets act on plain, commercially pure-titanium (*cp*-Ti) plates, we evaluated the distribution of biomolecules by immunofluorescence. PPARγ, PDGF-B, and TGFβ1 were similarly released at immunofluorescence levels from activated adherent platelets, retained in the surrounding extra-platelet spaces for a while, and did not immediately diffuse away to distant spaces. Exogenously added CaCl_2_ augmented release and retention of those biomolecules along with activation and aggregation. Taken together with our previous data regarding platelet adhesion, these findings suggest that especially when treated with CaCl_2_, platelets immediately adhere on *cp*-Ti plates to release their stored biomolecules in the absence of plasma proteins and that these biomolecules do not diffuse away, but stay longer in extra-platelet spaces around the platelets by newly formed, immature fibrin fiber fragments. Consequently, these retained biomolecules are anticipated to cooperatively stabilize implants by stimulating alveolar bone regeneration and integration.

## 1. Introduction

Among the various materials, titanium has been used as the most favorable material for dental implants [1,2,3]. This is based on the advantages that titanium has, such as relatively higher corrosion resistance and higher bio-inertness and bio-affinity. However, the bio-inertness is hardly anticipated to augment bio-integration between titanium implants and the surrounding soft and bone tissues. In the past two decades, many efforts, mainly on the industrial side, have been made to overcome this possible shortcoming and improve bio-integration, especially osseointegration. For example, the titanium surface has been modified micro-topographically or chemically by etching, blasting, ultraviolet (UV) ray irradiation, atmospheric pressure plasma (APP) treatment, and many other methods [4,5,6,7,8,9,10]. Furthermore, hybridization techniques with other materials, such as calcium phosphates, were recently introduced to improve the bio-affinity of implants to bone. The hybrid implants have been increasingly accepted by many clinical implantologists.

On the other hand, the end users of dental implants, clinical implantologists, have also independently attempted to modify the surface property by immersing or coating titanium implants with blood, platelet-rich plasma (PRP) [11,12,13,14,15,16,17,18], and therapeutic agents for periodontal regeneration, such as enamel matrix proteins. In such treatments, the titanium surface is commonly anticipated to obtain a higher bio-affinity and ultimately tighter or complete osseointegration by promoting the recruitment and proliferation of stem or progenitor cells involved in alveolar bone regeneration. However, there has been no scientific evidence to strongly support such chairside surface treatment prior to the embedment of dental implants.

To investigate the interaction between the titanium surface and the PRP, especially the platelets, and validate the use of PRP for surface modification, in a previous study [19], we identified the major adhesion molecules involved in human platelet adhesion and activation on commercially pure titanium (*cp*-Ti) plates. To further investigate these interactions, in this study, we visualized the distribution of the growth factors, PDGF-B and TGFβ1, and the transcription factor, peroxisome proliferator-activated receptor γ (PPARγ), which is recently thought to be an anti-inflammatory factor [20,21], and evaluated the biomolecule releasing activity of platelets activated and adhered on *cp*-Ti plates.

## 2. Materials and Methods

### 2.1. Preparation of PRP and Platelet Suspension in PBS

As described previously [19], ~9 mL of peripheral blood was collected from non-smoking healthy male volunteers (N = 6; age: 23 to 58 years old) in plain plastic blood-collection tubes (Neotube; NIPRO, Osaka, Japan) containing 1 mL of A formulation of acid-citrate-dextrose (ACD-A; Terumo, Tokyo, Japan) [22,23]. These blood samples were stored in a rotating agitator at ambient temperature until use (~48 h) [24,25]. PRP was prepared by the double-spin method: soft spin (472× *g* for 10 min) and subsequent hard spin (1065× *g* for 5 min).

After the initial soft spin using a centrifuge equipped with a swing rotor (KS-5000; Kubota, Tokyo, Japan), the upper plasma fraction was treated with 5 μg/mL of prostaglandin E_1_ (PGE_1_; Cayman Chemical Co., Ann Arbor, MI, USA) for 10 min to minimize the platelet aggregation. The samples were subjected to hard spin using a centrifuge equipped with an angle rotor (1–14; Sigma Laborzentrifugen GmbH, Osterode am Harz, Germany). Precipitated platelets were gently resuspended in PBS at appropriate concentrations (2.2–2.8 × 10^5^/µL) using an automated hematology analyzer (pocH 100iV; Sysmex, Kobe, Japan).

The study design and consent forms for all procedures (project identification code: 2297) were approved by the ethics committee for human participants at the Niigata University School of Medicine (Niigata, Japan) and complied with the Helsinki Declaration of 1964, as revised in 2013.

### 2.2. cp-Ti Plates and APP Treatment

The plain *cp*-Ti plates (Nilaco, Tokyo, Japan) were used in this study: the *cp*-Ti plates were cut into square 10 × 10 mm^2^ pieces, washed serially with acetone, ethanol, and distilled water in an ultrasonic cleaner (Citizen, Tokyo, Japan) and air-dried [19].

A non-thermal APP device (P500-SM; Sakigake-Semiconductor Co., Kyoto, Japan) was used in this study, as described previously [26]. Nitrogen was introduced as a feed gas at a rate of 5 L/min. To maximize the effects of the APP treatment, the *cp*-Ti plates were placed at a distance of 5 mm or less from the jet-nozzle and treated for 1 min.

### 2.3. Platelet Inoculation onto cp-Ti Plates

Platelet suspensions were inoculated onto the *cp*-Ti plates, and the plates were incubated at ambient temperature for up to 30 min. The *cp*-Ti plates were directly fixed with 10% neutralized formalin solution (Wako, Osaka, Japan) or ThromboFix (Beckman-Coulter, Atlanta GA, USA). Alternatively, the *cp*-Ti plates were vigorously washed two times with PBS on a shaker (~10 s) prior to fixation.

For activation, platelets in PBS suspensions were treated with 0.1% CaCl_2_, as described in previous studies [22,23].

### 2.4. Immunocytochemical Fluorescence Staining

Fixed platelets on the *cp*-Ti plates were directly subjected to treatment with primary antibodies, such as mouse monoclonal anti-CD62P antibody (1:20 dilution; BioLegend, San Diego, CA, USA), rabbit polyclonal anti-PDGF-B (1:200 dilution; Santa Cruz Biotechnology, Inc., Dallas, TX, USA), anti-TGFβ1 (1:200 dilution; Santa Cruz Biotechnology), rabbit monoclonal anti-PPARγ (1:100 dilution; Cell Signaling Technology, Danvers, MA, USA), and mouse monoclonal anti-fibrin antibodies (1:400 dilution; GeneTex, Inc., Irvine, CA, USA) overnight at 4 °C. In this study, based on our preliminary observations that our routine blocking or washing with Tween-20-containing PBS also visualized intra-platelet binding of the antibodies we used, we did not perform blocking or use such a detergent-containing PBS in the main experiments in this study.

The samples were then probed with the corresponding secondary antibodies or non-immunized rabbit IgG (Life Technologies Corporation, Carlsbad, CA, USA) as an isotype control for 60 min at ambient temperature in the dark. The samples were finally mounted using an antifade mounting medium (Vectashield; Vector Laboratories, Burlingame, CA, USA) and examined under a fluorescence microscope (ECLIPSE 80i; Nikon) connected with a cooled CCD camera (VB-7000; Keyence) [23].

### 2.5. Observation of Microparticles by Scanning Electron Microscopy (SEM)

Because of the difficulty of microparticle visualization on *cp*-Ti plates by SEM, the platelets were suspended in PBS and stimulated with 0.1% CaCl_2_ in polypropylene sample tubes and immediately transferred onto specific filters (Sem Pore; JOEL, Akishima, Japan). After 10-min incubation, the platelets were fixed with 2.5% neutralized glutaraldehyde, serially dehydrated, and freeze dried.

These samples were examined under a scanning electron microscope (TM-1000; Hitachi, Tokyo, Japan), as described previously [23,27].

### 2.6. Image Analyses of Immunoflurescence Photomicrographs

We randomly selected original, non-trimmed photomicrographs from representative single experiments (N = 4) and analyzed the images using the Winroof software (Mitani Co., Tokyo, Japan). In brief, the merged images were separated into three independent RGB images. Then, the CD62P^+^ and biomolecules^+^ regions in red and green, respectively, were determined as total pixels. The levels of released biomolecules were assessed by the ratio of biomolecules to CD62P.

### 2.7. Statistical Analysis

Data are represented in box plots. SigmaPlot (SigmaPlot 13.0; Systat Software, Inc., San Jose, CA, USA) evaluated the image analysis data as parametric by both normality and equal variance testing, and suggested a one-way ANOVA followed by Bonferroni’s multiple-comparisons test as the appropriate analysis. The results of this analysis, however, showed significant differences in both PPARγ and PDGF-B and therefore as these data were derived from a limited number of samples (N = 4), a non-parametric analysis was performed. Therefore, we compared the mean values via the Kruskal–Wallis one-way analysis of variance, followed by a Steel-Dwass multiple comparisons test (BellCurve for Excel; Social Survey Research Information Co., Ltd., Tokyo, Japan). Differences with *p* < 0.05 were considered statistically significant.

We obtained whole-blood samples from six volunteers. For practical reasons, we used at least four samples in each experiment; this discrepancy was not because of either opt-out by volunteers or exclusion of outliers.

## 3. Results

In this study, we chose to visualize not only the representative growth factors, i.e., PDGF-B and TGFβ1, but also a transcription factor, i.e., PPARγ, because the anti-inflammatory effects of PRP are recently thought to be mediated by PPARγ [20,21,28].

### 3.1. Optimization of Experimental Procedures

Because visualization of platelet-derived biomolecules was preliminarily found to be substantially influenced by experimental procedures, we began with a demonstration of the procedure-dependent differences in visualized platelet-derived biomolecules.

**Figure 1:**
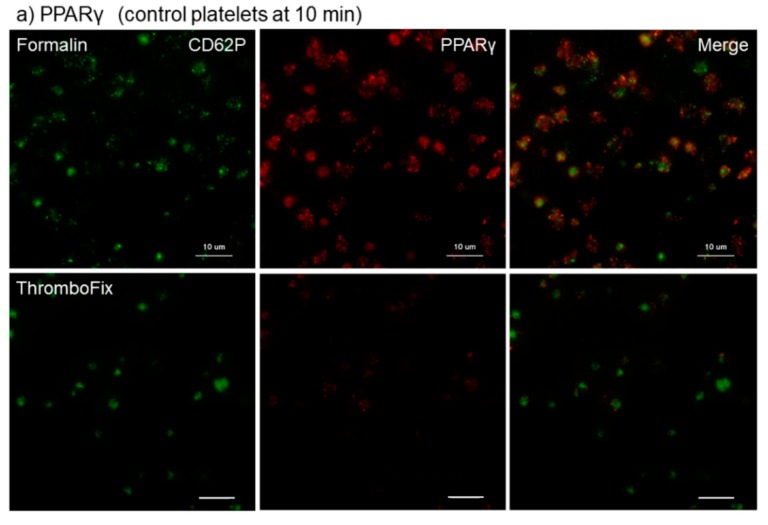

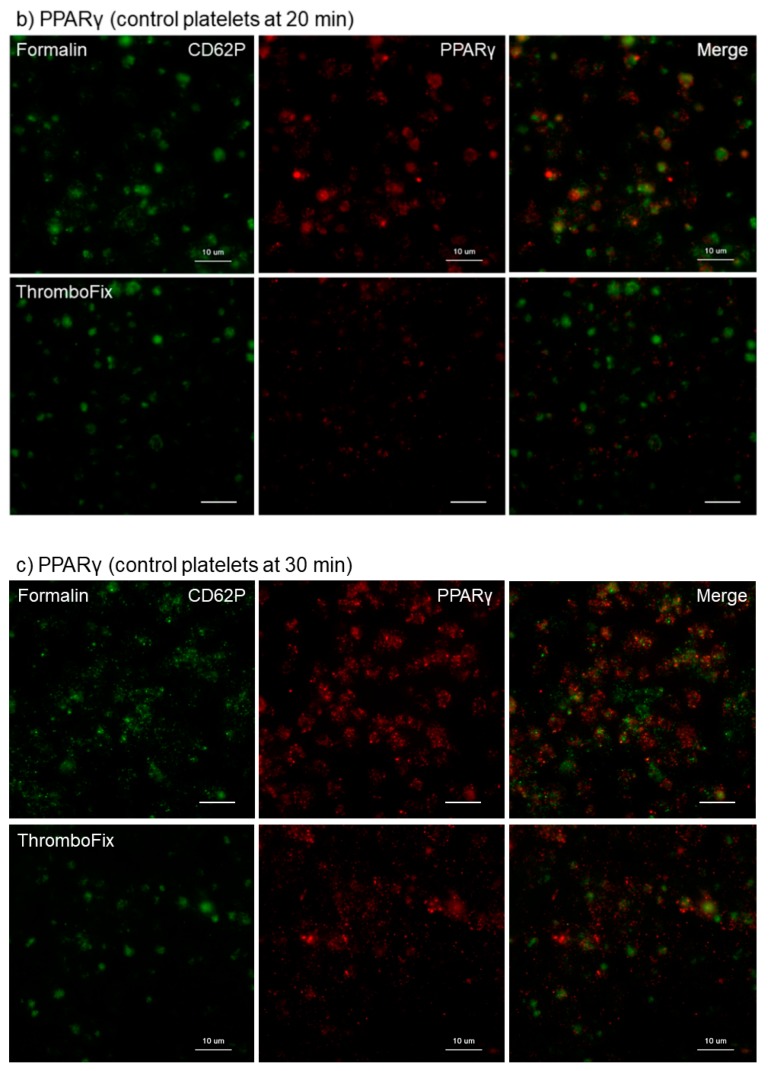
Different effects of fixatives on visualization of PPARγ in control platelets adhered onto cp-Ti plates. Platelets suspended in PBS were incubated on *cp*-Ti plates for (**a**) 10 min, (**b**) 20 min, or (**c**) 30 min and fixed with 10% neutral buffered formalin or ThromboFix without intensive washing. Activated platelets (CD62P; green) and PPARγ (red) were visualized by immunofluorescence and their distribution were compared between formalin and ThromboFix. Similar findings were obtained from the samples prepared from the other three donor samples, which were kept separate and tested individually. Bar = 10 µm.

The different effects of the fixatives 10% neutral buffered formalin and ThromboFix on the visualization of PPARγ in control platelets adhered onto *cp*-Ti plates at 10–30 min are shown in Figure 1. The specimens were double-stained for CD62P and PPARγ and both images were merged to identify the distribution of PPARγ. Although the levels varied with individuals, regardless of fixative types, almost all adherent platelets were visually positive for CD62P. In PPARγ-staining, the cytoplasm of almost all platelets was clearly positive following fixation with formalin, but not after fixation with ThromboFix. Instead, extra-platelet spaces became weakly, but widely, positively stained in a time-dependent manner.

**Figure 2:**
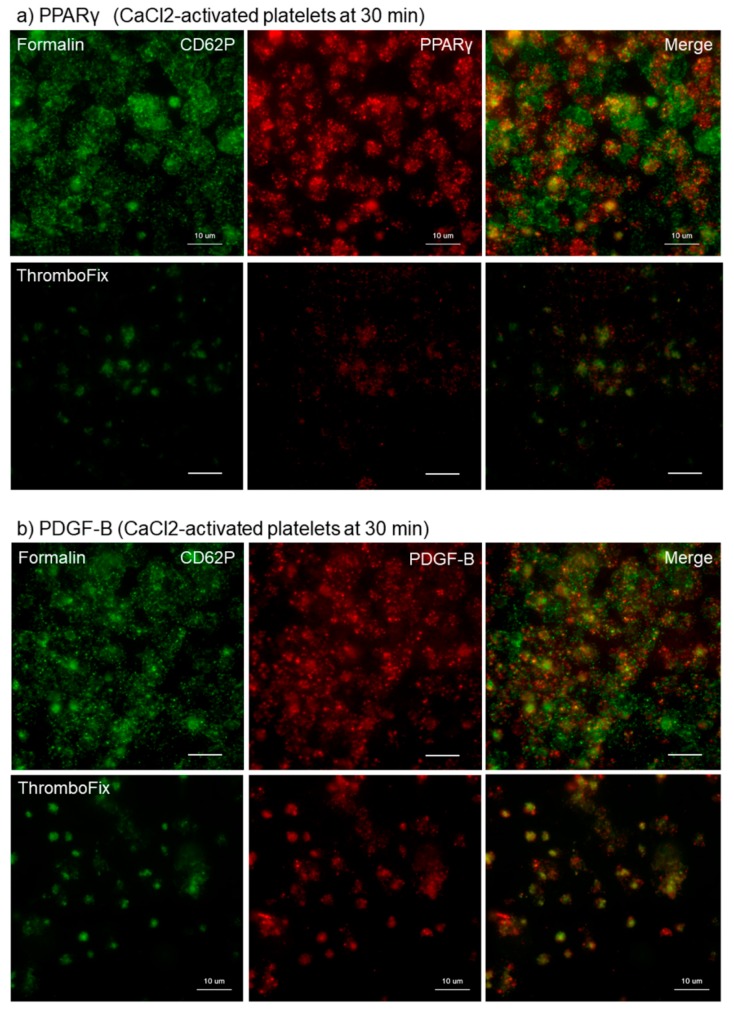
Different effects of fixatives on visualization of PPARγ and PDGF-B in Ca^2+^-activated platelets aggregated and adhered onto *cp*-Ti plates. Platelets were treated with 0.1% CaCl_2_ in PBS, incubated on *cp*-Ti plates for 30 min and fixed with 10% neutral buffered formalin or ThromboFix without intensive washing. (**a**,**b**) Activated platelets (CD62P; green), (**a**) PPARγ (red), and (**b**) PDGF-B (red) were visualized by immunofluorescence. Similar findings were obtained from the samples prepared from the other three donors. Bar = 10 µm.

This finding was confirmed for growth factors in Ca^2+^-activated platelets. The different effects of fixatives on visualization of PPARγ and PDGF-B in Ca^2+^-activated platelets aggregated and adhered onto *cp*-Ti plates at 30 min are shown in Figure 2. Essentially as shown in Figure 1, both PPARγ and PDGF-B were found at higher levels in the cytoplasm of platelets fixed with formalin than with ThromboFix. In ThromboFix-fixed platelets, extra-platelet spaces were widely positive.

**Figure 3:**
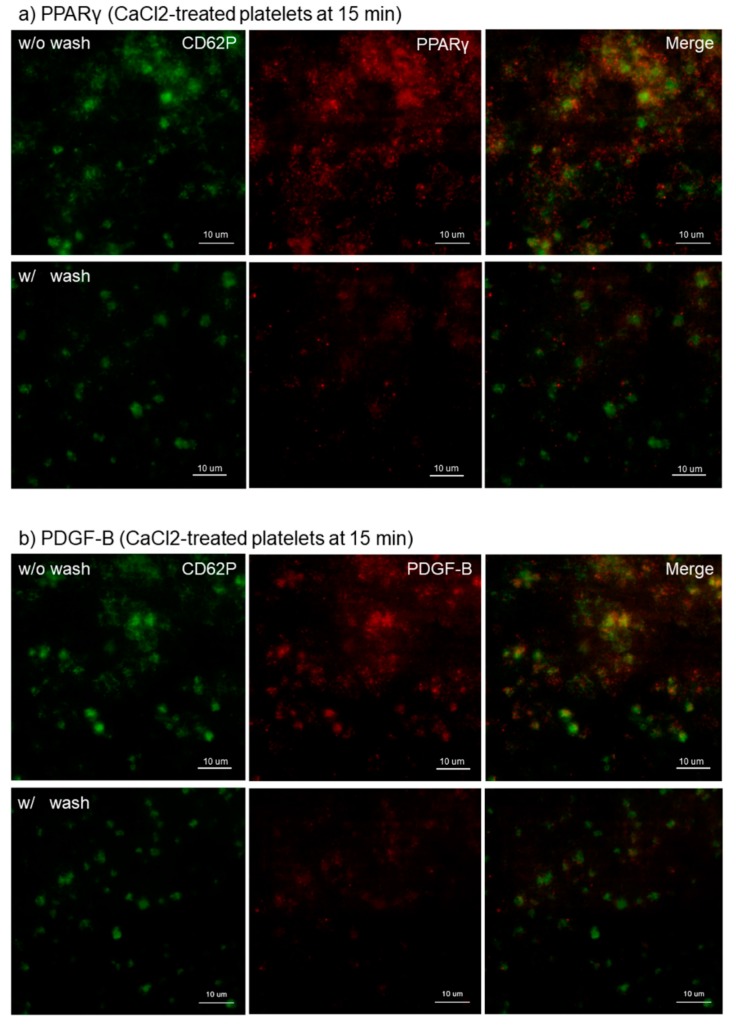

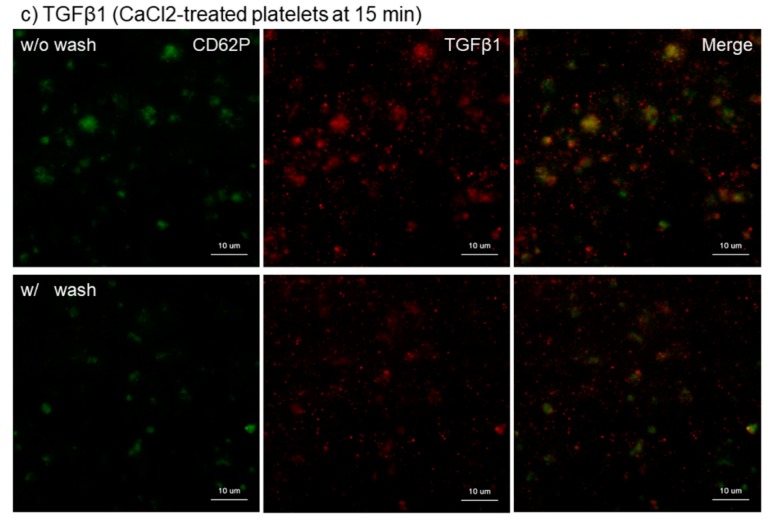
Effects of intensive washing on distribution of PPARγ, PDGF-B, and TBGβ1 in Ca^2+^-activated platelets on *cp*-Ti plates. Platelets were treated with 0.1% CaCl_2_ in PBS, incubated on *cp*-Ti plates for 15 min and fixed with ThromboFix with or without intensive washing. (**a**–**c**) Activated platelets (CD62P; green), (**a**) PPARγ (red), (**b**) PDGF-B (red), and (**c**) TGFβ1 (red) were visualized by immunofluorescence. Similar findings were obtained from the samples prepared from the other three donors. Bar = 10 µm.

The effects of intensive washing on the distribution of PPARγ, PDGF-B, and TGFβ1 in Ca^2+^-activated platelets at 15 min of fixation with ThromboFix are shown in Figure 3. Regardless of platelet-derived biomolecule types, the intensive washing totally reduced those biomolecules by partially excluding fibrin fiber fragments and probably also weakly adherent platelets without apparently influencing CD62P expression in strongly adherent platelets.

**Figure 4:**
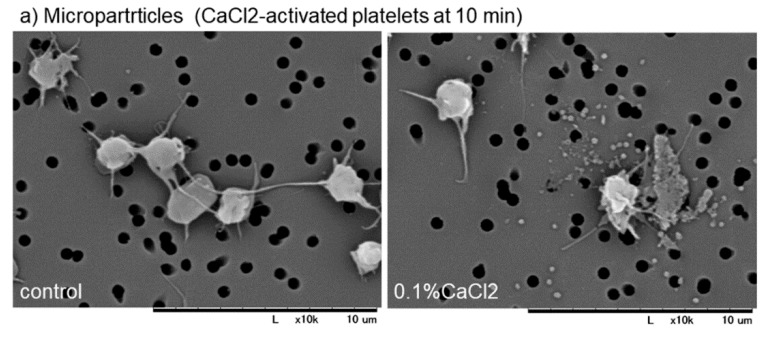

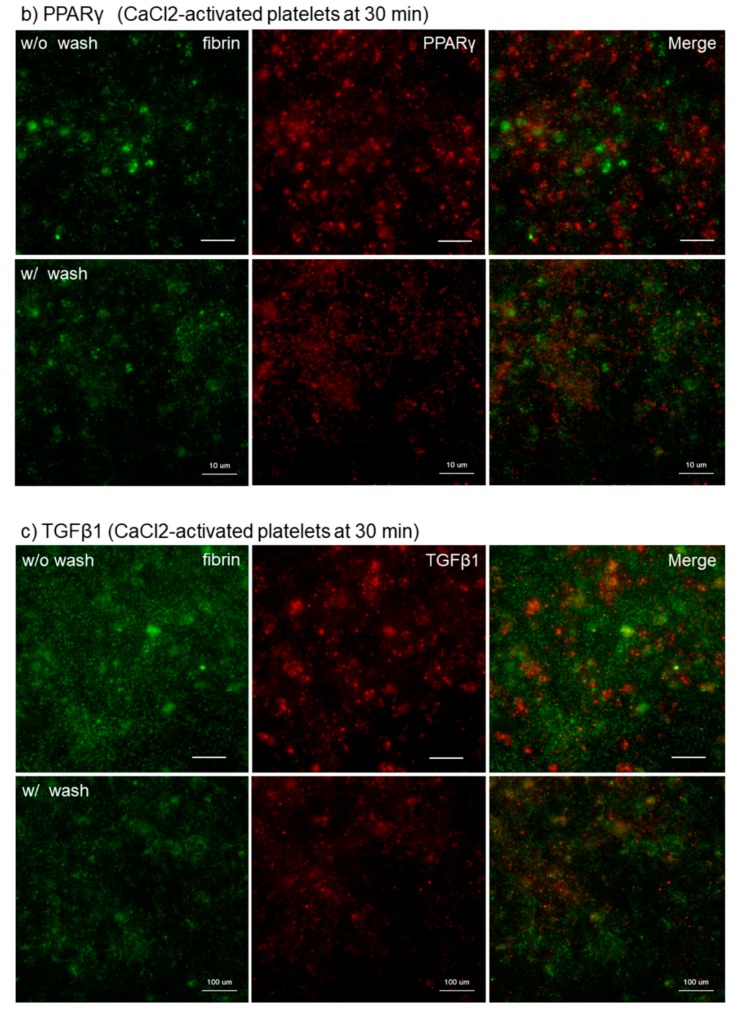
(**a**) Effects of activation with CaCl_2_ on microparticle release and (**b**,**c**) effects of intensive washing on the levels of fibrin formed on and around platelets. (**a**) Platelets suspended in PBS were activated by 0.1% CaCl_2_, incubated on the filter for 10 min, fixed with glutaraldehyde, and subjected to SEM examination. (**b**,**c**) As described in the legend of Figure 3, activated platelets were fixed with or without intensive washing and (**b**,**c**) Activated platelets (CD62P; green), (**b**) PPARγ (red), and (**c**) TGFβ1 (red) were visualized by immunofluorescence. Similar findings were obtained from the samples prepared from the other three donors. Bar = 10 µm.

To support this finding, the distribution of microparticles and fibrin fiber fragments need to be examined. Effects of activation with CaCl_2_ on microparticle release and effects of intensive washing on the levels of fibrin formed on and around platelets are shown in Figure 4. Because microparticles, if any, are embedded with plasma proteins after preparation for SEM examination, the presence of microparticles was examined on the specific filter after activation with 0.1% CaCl_2_. In Ca^2+^-activated platelets, many microparticles were released 10 min after activation (Figure 4a). Intensive washing totally reduced the apparent amounts of fibrin fiber fragments and simultaneously those of PPARγ and TGFβ1 in Ca^2+^-activated platelets at 30 min (Figure 4b,c).

### 3.2. Effects of APP and CaCl_2_ on Platelet-Derived Factor Release

According to the results obtained by the optimization of experimental procedures, we adjusted the preparation protocol so that platelets adhered on *cp*-Ti plates are fixed with ThromboFix without receiving intensive washing in the following experiments. In addition to the treatment of platelets with 0.1% CaCl_2_, *cp*-Ti plates were treated with non-thermal APP immediately prior to the inoculation of platelets. This was based on our working hypothesis that APP-treatment facilitates platelet adhesion and activation and thereby augments the release of platelet-derived biomolecules.

**Figure 5:**
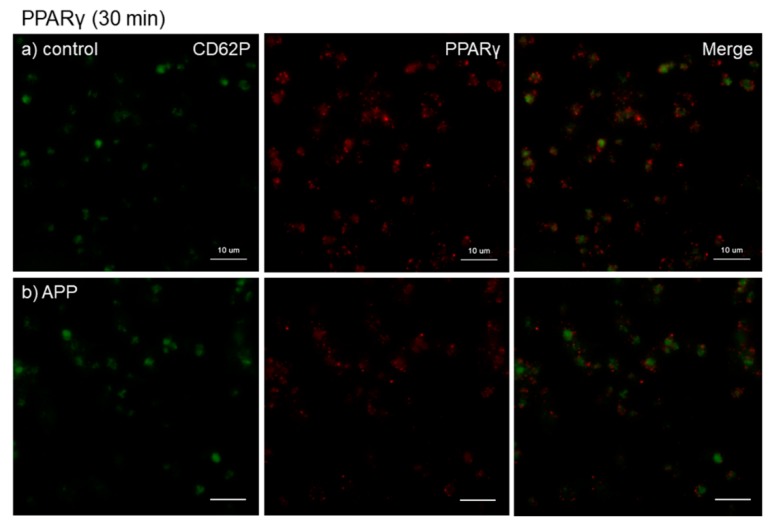

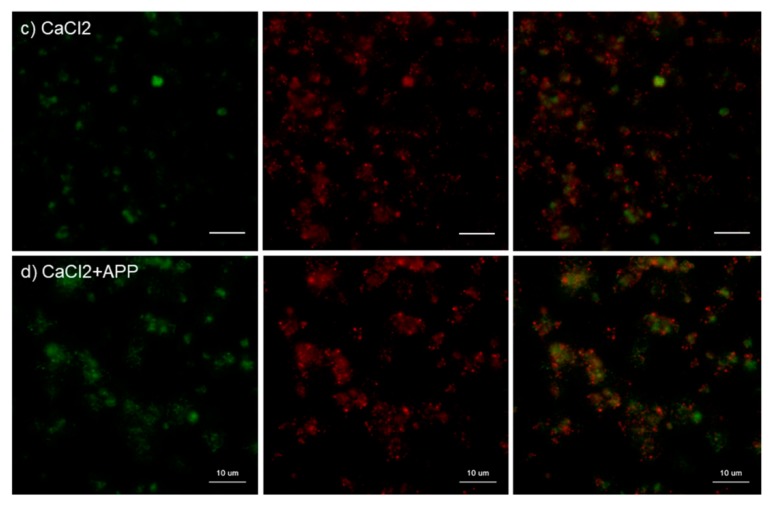
Effects of CaCl_2_ and atmospheric pressure plasma (APP) treatments on PPARγ distribution on *cp*-Ti plates at 30 min. Platelets were prepared, treated, and fixed for visualization (green: CD62P, red: PPARγ) as described in the legend of Figure 4. (**a**) Control platelets on non-treated *cp*-Ti plates, (**b**) control platelets on APP-treated *cp*-Ti plates, (**c**) CaCl_2_-stimulated platelets on non-treated *cp*-Ti plates, (**d**) CaCl_2_-stimulated platelets on APP-treated *cp*-Ti plates. Similar findings were obtained from the samples prepared from the other three donors. Bar = 10 µm.

The effects of CaCl_2_ and APP treatments on PPARγ distribution on *cp*-Ti plates at 30 min are shown in Figure 5. Again, CaCl_2_ substantially increased PPARγ release and/or retention in extra-platelet spaces, while APP significantly reduced the basal and CaCl_2_-induced increases in PPARγ release and/or retention. These merged photomicrographs were image-analyzed and the quantified results are shown in Figure S1a. However, no statistical differences were observed among these groups according to non-parametric analyses.

**Figure 6:**
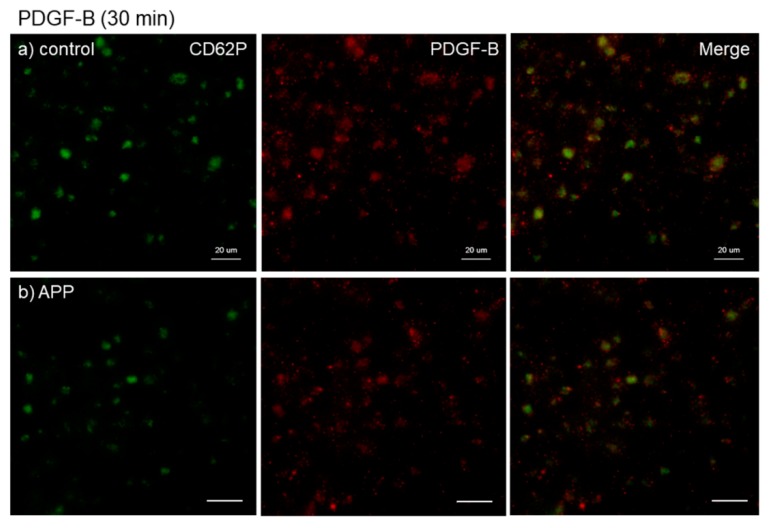

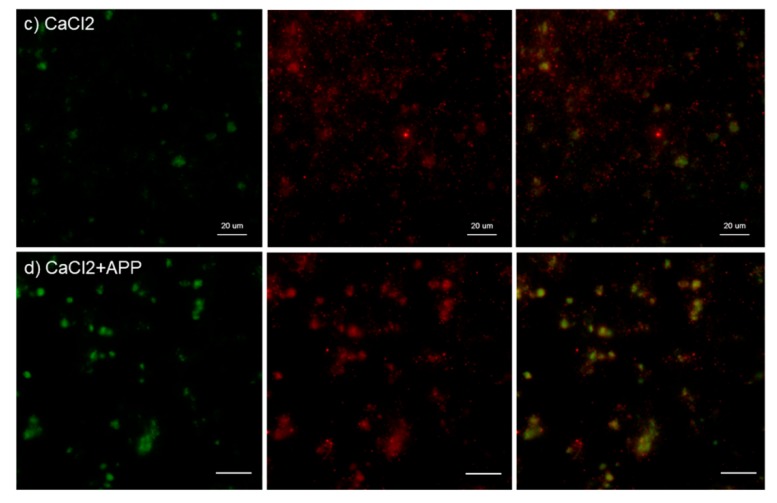
Effects of CaCl_2_ and APP treatments on PDGF-B distribution on *cp*-Ti plates at 30 min. Platelets were prepared, treated, and fixed for visualization (green: CD62P, red: PDGF-B) as described in the legend of Figure 4. (**a**) Control platelets on non-treated *cp*-Ti plates, (**b**) control platelets on APP-treated *cp*-Ti plates, (**c**) CaCl_2_-stimulated platelets on non-treated *cp*-Ti plates, (**d**) CaCl_2_-stimulated platelets on APP-treated *cp*-Ti plates. Similar findings were obtained from the samples prepared from the other three donors. Bar = 10 µm.

The effects of CaCl_2_ and APP treatments on PDGF-B distribution on *cp*-Ti plates at 30 min are shown in Figure 6. CaCl_2_ appeared to increase PDGF-B release and/or retention in extra-platelet spaces, whereas APP to some extent reduced the CaCl_2_-induced increases in PDGF-B release and/or retention. These merged photomicrographs were image-analyzed and the quantified results are shown in Figure S1b. Again, no statistical differences were observed among these groups according to non-parametric analyses.

**Figure 7:**
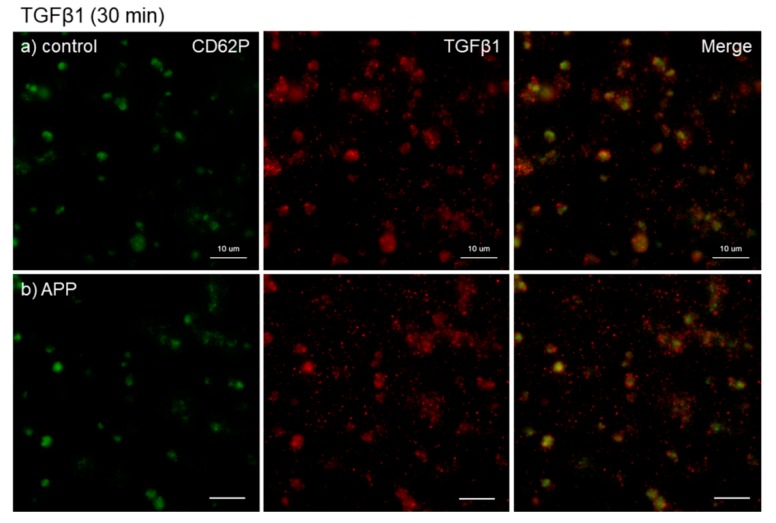

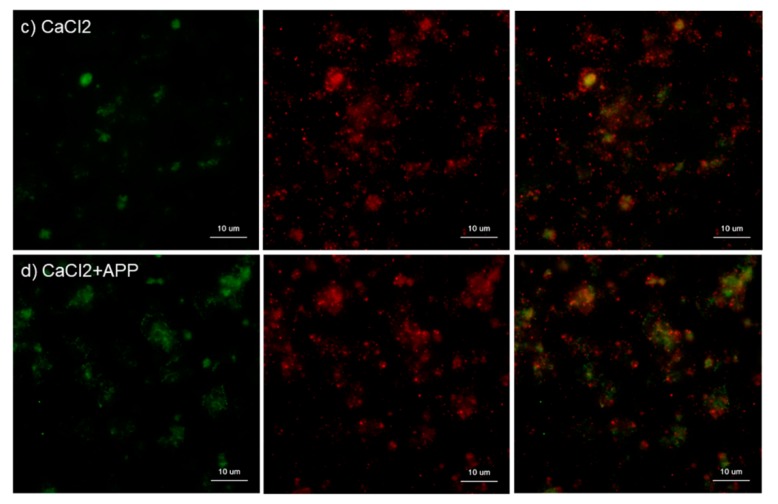
Effects of CaCl_2_, and APP treatments on TGFβ1 distribution on cp-Ti plates at 30 min. Platelets were prepared, treated, and fixed for visualization (green: CD62P, red: TGFβ1) as described in the legend of Figure 4. (**a**) Control platelets on non-treated cp-Ti plates, (**b**) control platelets on APP-treated cp-Ti plates, (**c**) CaCl_2_-stimulated platelets on non-treated cp-Ti plates, (**d**) CaCl_2_-stimulated platelets on APP-treated cp-Ti plates. Similar findings were obtained from the samples prepared from the other three donors. Bar = 10 µm.

The effects of CaCl_2_ and APP treatments on TGFβ1 distribution on *cp*-Ti plates at 30 min are shown in Figure 7. CaCl_2_ slightly increased TGFβ1 release and/or retention in extra-platelet spaces. APP to some extent reduced the CaCl_2_-induced TGFβ1 release and/or retention. These merged photomicrographs were image-analyzed and the quantified results are shown in Figure S1c. Again, no statistical differences were observed among these groups according to non-parametric analyses.

## 4. Discussion

It is generally accepted that in the process of wound-healing, platelets gather and aggregate at injury sites and release growth factors and other soluble biomolecules from α-granules to extra-platelet spaces occupied mainly by fibrin fibers, a complex which is called a blood clot [29,30]. Thus, growth factors released from platelets do not immediately diffuse away but can stay there for a while to induce blood capillaries and stem and progenitor cells involved in tissue repair and regeneration. In addition, anti-inflammatory factors, including PPARγ, simultaneously released from activated platelets [31], are expected to suppress inflammation and indirectly facilitate tissue repair and regeneration in cooperation with growth factors such as PDGF and TGFβ [32].

On the basis of these physiological functions of platelets, PRP and its derivatives have been developed and applied in regenerative therapy. In the case of its application in pretreatment of dental implants, PRP is expected to stimulate the growth and adhesion of the surrounding osteoblastic and other cell types in connective tissues and ultimately improve the stability and osseointegration of embedded implants. PDGF, TGFβ, and PPARγ have been often demonstrated to have potential positive effects. PDGF enhances bone formation and bone-to-implant contact through direct activation of osteoblasts, in addition to recruitment of mesenchymal stem cells through vascularization [33], and consequently accelerates the process of osseointegration [34,35,36]. Although being unable to directly induce osteogenesis in stem cells, TGFβ1 increases the pool of osteoprogenitors by inducing chemotaxis and proliferation [37]. These actions may eventually contribute to early implant stability. In addition, it is generally thought that inflammation exerts negative effects on osseointegration of Ti-based implants [38]. Lee et al. [39] have recently demonstrated in the diabetes mellitus-induced rat model that PPARγ released from the corresponding cDNA introduced on titanium implant surfaces enhances osseointegration and implant longevity. However, there are limited data that supports such an application of PRP.

**Figure 8:**
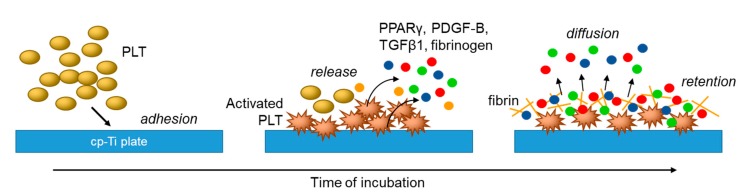
Proposed mechanisms of platelet adhesion and activation and subsequent growth factor release and retention on *cp*-Ti plates.

In the previous study [19], we demonstrated the time-dependent adhesion of platelets onto the *cp*-Ti platelets and the adhesion molecules mainly involved in this initial adhesion. To add new findings to the previous data, this study was performed to visualize the release of growth factors and a transcription factor. Although not determining those soluble biomolecules in a quantitative manner, this immunofluorescence method has a merit to semi-quantitatively demonstrate those distribution and level on a small scale of sample without procedure-dependent loss of soluble biomolecules. In a previous study regarding PRF matrix [32], we successfully demonstrated distribution of growth factors by the immunohistochemical method. The main finding of this study is, as illustrated in Figure 8, that in the absence of plasma proteins such as fibrinogen and albumin that disturb platelet adhesion, platelets immediately adhere to *cp*-Ti plates and become mildly activated to release PDGF-B, TGFβ1, and PPARγ to extra-platelet spaces. Additional stimuli such as exogenously added Ca^2+^ to facilitate platelet adhesion and activate further release of growth factors [23]. These biomolecules are then retained by newly formed fibrin fragments adjacent to activated platelets on *cp*-Ti plates. The other possible, but minor, mechanism is that growth factors may be captured by their specific receptors expressed on the membrane surface of the platelets [40].

Our data for optimization of experimental procedures suggest that the routine fixation method using 10% formalin is not suitable to visualize only platelet-derived biomolecules distributed in extra-platelet spaces. According to the manufacturer’s document, formalin solution we used contains 5–10% methanol as a stabilizer. Thus, “10% neutralized formalin solution” is calculated to contain 0.5–1.0% methanol. This amount of methanol is sufficient to perforate the plasma membrane of the platelets and platelet-derived biomolecules stored in α-granules could be visualized even without treatment with specific detergents. The formula of the fixative we chose, ThromboFix, is not disclosed by the manufacturer; however, considering its formula is optimized for evaluation of platelet CD62P by flow-cytometry, we speculate that this fixative stabilizes platelets without chemically damaging the integrity of the membrane of the platelets and thereby that the intra-platelet growth and transcription factors could be masked from immunofluorescence detection.

Because platelets contain and release fibrinogen and thrombin upon activation, it was theoretically difficult to exclude the possible involvement of fibrin formation in retention of platelet-derived biomolecules. However, we preliminarily found that intensive washing with tapping reduced the amount of immature fibrin formed around the platelets and applied this procedure to test the possible retention of growth factors by the fibrin matrix. Although not completely excluding fibrin, this washing process apparently reduced the platelet-derived biomolecules distributed around the adherent platelets. Therefore, these findings support the hypothesis that platelet-derived biomolecules released are trapped and retained in the surrounding extra-platelet spaces occupied mainly with immature fibrin fragments. However, it is also possible that those biomolecules are to some extent adsorbed onto the *cp*-Ti surface and platelet membrane receptors.

Regarding the effects of APP treatment, it is known that APP treatment enhances adhesive quality, fibrin polymerization capacity, and surface coating efficiency [41]. Therefore, this technology has been applied in the modification of dental implant surfaces. In this study, we confirmed that APP treatment markedly increased the surface wettability [26]. Thus, we initially raised a working hypothesis that APP treatment makes platelet adhesion on *cp*-Ti plates more rapid and tighter, as shown previously in osteoblastic progenitor cells [26]. Contrary to our expectation, however, APP treatment showed no positive effects on platelet adhesion and fibrin polymerization in this study. Exceptionally, APP treatment significantly reduced the distribution of all the platelet-derived biomolecules in Ca^2+^-activated platelets. The most likely explanation for this phenomenon is that platelet-derived biomolecules diffuse away rapidly. However, it cannot be ruled out that platelet-derived biomolecules could not be visualized clearly in a single focal plane because of the thicker polymerized fibrin in the APP-treated *cp*-Ti plates than in the control plates. This is a limitation of a regular fluorescent microscope. The possible effects of APP treatment on fibrin polymerization on *cp*-Ti plates should be further investigated with a confocal microscope or other powerful modern modalities for 3D visualization.

## 5. Conclusions

The present findings suggest that within the limitation of the study that the pretreatment of dental implants with plasma-free platelet suspension increases the abundance of platelets, growth factors, and the anti-inflammatory factor PPARγ, which could contribute to the improved initial stability and osseointegration of titanium dental implants.

## Supplementary Materials

The following are available online at https://www.mdpi.com/2304-6767/7/4/109/s1, Figure S1: Analyses of immunofluorescence photomicrograph images of CD62P and other biomolecules (a: PPARγ, b: PDGF-B, c: TGFβ1). Each photograph was separated into three RGB files and the individual regions or particles positive for CD62P or the biomolecules were expressed as total pixels. The ratio of each biomolecule to CD62P is expressed as a box plot. N = 4.

## Abbreviations

PDGF-B	platelet-derived growth factor-B
TGFβ1	transforming growth factor β1
PPARγ	peroxisome proliferator-activated receptor γ
PRP	platelet-rich plasma
*cp*-Ti	commercially pure-titanium
APP	atmospheric pressure plasma
UV	ultraviolet
PGE_1_	prostaglandin E_1_
PBS	Phosphate buffer saline
IgG	immunoglobulin
