# Supplementary material for: Platelet Adhesion on Commercially Pure Titanium Plates in Vitro II. Immunofluorescence Visualization of PDGF-B, TGFβ1, and PPARγ Released from Activated Adherent Platelets
Source: Dent J (Basel). 2019 Nov 19;7(4):109. doi: 10.3390/dj7040109 (PMC6960926; doi:10.3390/dj7040109)
Supplement: Supplementary file 1 [file dentistry-07-00109-s001.pdf]

Supplementary Materials: Platelet Adhesion on Commercially Pure Titanium Plates in Vitro II. Immunofluorescence Visualization of PDGF-B, TGFβ1, and PPARγ Released from Activated Adherent Platelets

Tetsuhiro Tsujino, Akira Takahashi, Taisuke Watanabe, Kazushige Isobe, Yutaka Kitamura, Kazuhiro Okuda, Koh Nakata and Tomoyuki Kawase

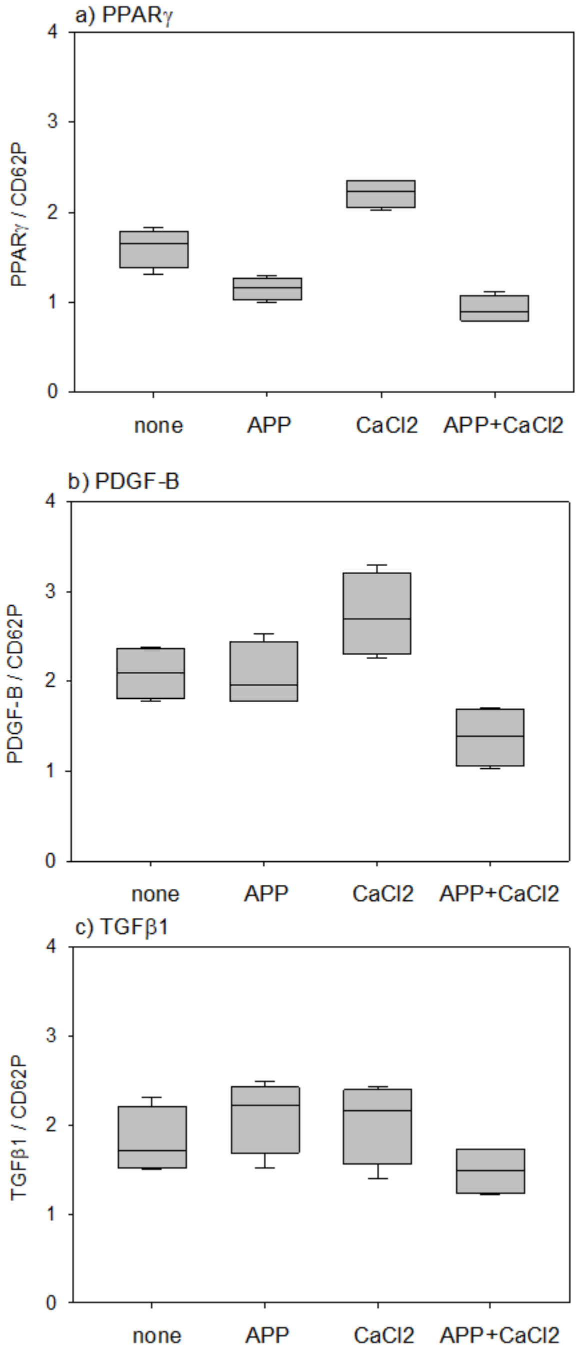

Figure S1. Analyses of immunofluorescence photomicrograph images of CD62P and other biomolecules (a: PPARγ, b: PDGF-B, c: TGFβ1). Each photograph was separated into three RGB files and the individual regions or particles positive for CD62P or the biomolecules were expressed as total pixels. The ratio of each biomolecule to CD62P is expressed as a box plot. N = 4
